# Stress in paradise: effects of elevated corticosterone on immunity and avian malaria resilience in a Hawaiian passerine

**DOI:** 10.1242/jeb.242951

**Published:** 2021-10-19

**Authors:** Gabrielle R. Names, Elizabeth M. Schultz, Jesse S. Krause, Thomas P. Hahn, John C. Wingfield, Molly Heal, Jamie M. Cornelius, Kirk C. Klasing, Kathleen E. Hunt

**Affiliations:** 1 Animal Behavior Graduate Group, University of California, Davis, One Shields Avenue, Davis, CA 95616, USA; 2 Department of Neurobiology, Physiology and Behavior, University of California, Davis, One Shields Avenue, Davis, CA 95616, USA; 3 Department of Biology, Wittenberg University, 200 W Ward Street, Springfield, OH 45504, USA; 4 Department of Biology, University of Nevada Reno, 1664 North Virginia Street, Reno, NV 89557, USA; 5 Department of Integrative Biology, Oregon State University, 2701 SW Campus Way, Corvallis, OR 97331, USA; 6 Department of Animal Science, University of California, Davis, One Shields Avenue, Davis, CA 95616, USA; 7 Smithsonian-Mason School of Conservation & Department of Biology, George Mason University, 1500 Remount Rd, Front Royal, VA 22630, USA

**Keywords:** *Plasmodium relictum*, Hawaiian honeycreeper, Innate immunity, Glucocorticoids, Introduced disease, Eco-physiology

## Abstract

Vertebrates confronted with challenging environments often experience an increase in circulating glucocorticoids, which result in morphological, physiological and behavioral changes that promote survival. However, chronically elevated glucocorticoids can suppress immunity, which may increase susceptibility to disease. Since the introduction of avian malaria to Hawaii a century ago, low-elevation populations of Hawaii Amakihi (*Chlorodrepanis virens*) have undergone strong selection by avian malaria and evolved increased resilience (the ability to recover from infection), while populations at high elevation with few vectors have not undergone selection and remain susceptible. We investigated how experimentally elevated corticosterone affects the ability of high- and low-elevation male Amakihi to cope with avian malaria by measuring innate immunity, hematocrit and malaria parasitemia. Corticosterone implants resulted in a decrease in hematocrit in high- and low-elevation birds but no changes to circulating natural antibodies or leukocytes. Overall, leukocyte count was higher in low- than in high-elevation birds. Malaria infections were detected in a subset of low-elevation birds. Infected individuals with corticosterone implants experienced a significant increase in circulating malaria parasites while untreated infected birds did not. Our results suggest that Amakihi innate immunity measured by natural antibodies and leukocytes is not sensitive to changes in corticosterone, and that high circulating corticosterone may reduce the ability of Amakihi to cope with infection via its effects on hematocrit and malaria parasite load. Understanding how glucocorticoids influence a host's ability to cope with introduced diseases provides new insight into the conservation of animals threatened by novel pathogens.

## Abstract (precis)

**Summary:** Amakihi innate immunity, as measured by natural antibodies and leukocytes, is not sensitive to changes in corticosterone, but high circulating corticosterone may reduce the ability of Amakihi to cope with avian malaria infection via its effects on hematocrit and malaria parasite load.

## INTRODUCTION

When vertebrates are challenged by acute environmental perturbations, the hypothalamic–pituitary–adrenal (HPA) axis is activated and several endocrine changes occur, including increased synthesis of glucocorticoid hormones. The surge in circulating glucocorticoids causes morphological, physiological and behavioral changes that promote survival during and after exposure to an environmental stressor (McEwen and Wingfield, 2003; Sapolsky et al., 2000; Wingfield et al., 1998). However, chronically high glucocorticoids can reduce fitness through effects on reproduction, growth, muscle mass and immunity (Dickens and Romero, 2013; Koutsos and Klasing, 2014; Romero and Wingfield, 2016; Sapolsky et al., 2000).

Corticosterone (CORT) is the primary glucocorticoid in birds, reptiles, adult amphibians and many rodents (Comendant et al., 2003; Gong et al., 2015; Narayan et al., 2013; Romero et al., 1998). Elevated CORT can cause immunosuppression or immunostimulation, depending on the duration of the change in CORT (Martin, 2009). Over short periods of time (minutes to hours), high CORT generally prepares the immune system for enhanced activity through immunostimulation (e.g. elevated cytokine expression, cell-mediated immune responses) and enhanced leukocyte trafficking (Dhabhar and McEwen, 1997; Koutsos and Klasing, 2014; Shini et al., 2010). In contrast, chronically high CORT (days to weeks) can cause immunosuppression in multiple taxa, including birds, by affecting humoral immunity (Bourgeon and Raclot, 2006; Stier et al., 2009), cell-mediated immunity (Dhabhar and McEwen, 1997; Titon et al., 2019), innate immunity (Kaiser et al., 2015) and immune organ size (Shini et al., 2009). Prolonged exposure to environmental challenges may increase susceptibility to disease if chronically elevated CORT results in immunosuppression (Dhondt and Dobson, 2017). In support of this hypothesis, West Nile virus mortality and parasite load were higher in passerine birds treated with exogenous CORT for several days compared with control individuals (Gervasi et al., 2017; Owen et al., 2012), and similar patterns have been described in other taxa (e.g. pygmy rattlesnakes, *Sistrurus miliarius*, Lind et al., 2018; red-legged salamanders, *Plethodon shermani*, Fonner et al., 2017).

Avian malaria is a widespread disease that infects species from nearly all avian taxa (Atkinson, 2008). Avian malaria can cause high mortality in endemic island birds (Pratt, 2009; Schoener et al., 2014), can reduce the fitness of continental birds through its effects on aging, offspring quality and mating behaviors (Asghar et al., 2015; Gilman et al., 2007), and has been implicated in recent declines of songbirds in Europe (Dadam et al., 2019). Some evidence suggests that elevated CORT can affect avian susceptibility to malaria. Free-living house sparrows (*Passer domesticus*) experience avian malaria relapse in the spring when their CORT levels are high, and malaria parasite loads increased in infected sparrows treated with CORT for 10 days (Applegate, 1970), although CORT levels before and after treatment were not measured in this study. Several days of elevated CORT can also result in decreased hematocrit (percentage of whole blood composed of erythrocytes; Beck et al., 2016) and low hematocrit during malaria infection lowers the oxygen-carrying capacity of the blood, which weakens the host and contributes to malaria mortalities (LaPointe et al., 2012). However, the evidence linking CORT to malaria susceptibility is limited, and the relationship remains wholly unexplored in novel avian malaria hosts.

Since the introduction of avian malaria to Hawaii in the early 1900s, the disease has contributed to declines of multiple Hawaiian honeycreeper species, a group of endemic songbirds that have evolved largely in the absence of blood parasites such as avian malaria (Pratt, 2009). Mortality rates have been particularly high at low elevations because in Hawaii, the avian malaria parasite (*Plasmodium relictum*) and its main vector, the southern house mosquito (*Culex quinquefasciatus*), are cold intolerant (LaPointe et al., 2010). As a result, robust vector populations and malaria transmission are supported year-round by warmer temperatures at low elevations (<1200 m above sea level, ASL), while infections are rare in cooler, high-elevation habitats (>1500 m ASL; LaPointe et al., 2012).

The Hawaii Amakihi (*Chlorodrepanis virens*, hereafter referred to as Amakihi) is the only honeycreeper that has maintained stable populations in both high- and low-elevation habitats of Hawaii (Samuel et al., 2015; Woodworth et al., 2005). Previous studies of Amakihi on Hawaii Island found that most low-elevation birds were infected with avian malaria, but mortality as a result of experimental infections was reduced in low- compared with high-elevation birds (Atkinson et al., 2013; Samuel et al., 2015), indicating that low­-elevation Amakihi are better able to cope with infection. When infected with avian malaria for the first time, honeycreepers experience an acute phase of infection, which causes a drop in hematocrit, decreased feeding, reduced activity and, sometimes, the death of the host (LaPointe et al., 2012). Amakihi that survive this phase subsequently experience a rapid drop in parasite load, but they maintain chronic, low levels of infection for life (Atkinson et al., 2001, 2013). These results suggest that strong disease pressures on low-elevation Amakihi have selected for increased resilience, i.e. the ability to recover following infection (Schneider, 2011; not to be confused with tolerance, i.e. the ability to support high parasite loads without severe illness or death; Richardson, 2016), rather than resistance, i.e. the ability to entirely eliminate pathogen burden (Atkinson et al., 2013; Eggert et al., 2008).

The objective of this study was to investigate how chronically elevated CORT affects innate immunity and hematological health of Amakihi that have experienced strong selection (low-elevation populations) and weak selection (high-elevation populations) by avian malaria on Hawaii Island. We hypothesized that CORT affects avian malaria resilience in Amakihi through its modulation of immunity and hematocrit. To test this hypothesis, we experimentally elevated CORT levels in high- and low-elevation adult (age 2+ years) male Amakihi for a period of 4 days using Silastic implants, and we measured the effect of CORT treatment on innate immunity and hematological health. We predicted that the CORT treatment would result in an increase in circulating baseline CORT levels and a decrease in immunity and hematocrit of both high- and low-elevation birds. Additionally, among birds infected with avian malaria, we predicted that CORT treatment would cause an increase in circulating malaria parasite load. To assess immunity, we utilized the hemolysis–hemagglutination assay (Matson et al., 2005) and we quantified circulating leukocytes, which reflect several components of innate immunity involved in controlling malaria infections (Artavanis-Tsakonas et al., 2003; Congdon et al., 1969; Cornet and Sorci, 2014; Lee et al., 2006). We determined hematocrit, which declines during acute malaria infection and can be affected by CORT, using centrifuged microhematocrit tubes, and we quantified *Plasmodium* parasitemia from blood smears.

## MATERIALS AND METHODS

### Study species and capture

The Amakihi, *Chlorodrepanis virens* (Gmelin 1788), is a small passerine endemic to the Hawaiian Islands (Sibley and Ahlquist, 1982). Amakihi are nectarivorous, non-migratory and relatively sedentary, and inhabit a wide range of habitats, including forests, shrublands and housing subdivisions from the coast to subalpine areas (Baldwin, 1953; Lindsey et al., 1998). Birds were captured from two high-elevation sites (>1500 m ASL) and one low-elevation site (<700 m ASL) on Hawaii Island (Fig. S1) using 3 m-high mist nets between 07:00 h and 17:00 h in March and April of 2019.

Sex and age were determined upon capture by visual inspection of plumage and the presence or absence of a brood patch, which develops only in females (Samuel et al., 2015). We restricted our study to adult males to increase our statistical power, because immunity and CORT can vary by sex and age (e.g. Names et al., 2021a; Vermeulen et al., 2017; Wilcoxen et al., 2011). Adult males received unique color combinations of plastic leg bands, their wing chord (to the nearest 0.1 mm using calipers) and mass (to the nearest 0.5 g using a 30 g Pesola scale) were measured, and their fat stores (furcular and abdominal) were scored on a scale of zero (lean) to five (fat; Kaiser, 1993). Birds were then hand-fed Nektar-Plus (artificial nectar high in sugars and supplemented with proteins, vitamins and minerals, Nekton Corporation, Keltern, Germany) and placed in temporary holding cages for transportation to the mosquito-proof aviary at Hawaii Volcanoes National Park, HI, USA, and their capture locations were recorded using a GPS unit (eTrex 20x, Garmin International Inc., Olathe, KS, USA) to facilitate release at their sites of capture following the conclusion of the experiment. Birds were captured, held and processed with the approval of the University of California Davis Institutional Animal Care and Use Committee (protocols 19297, 21082), the State of Hawaii Department of Land and Natural Resources (permits WL17-11, WL19-08, WL19-30, and Natural Area Reserves System and Forest Reserve access permits), the US Fish and Wildlife Service (permit MB62705C-1), the US Geological Survey Bird Banding Laboratory (permit 22712) and the US National Park Service (permit no. HAVO-2016-SCI-0027, HAVO-2018-SCI-0001, HAVO-2019-SCI-0008).

### Captive conditions and experimental design

High-elevation (*N*=20) and low-elevation (*N*=20) adult males were housed in individual cages (90×60×30 cm) located in one of two housing rooms (5.5×3.75×2.5 m) within the aviary. Each cage was equipped with multiple perches, supplemental heat (150 W ceramic heat lamps), Nektar-Plus (artificial nectar, detailed above) and several solid foods (orange, papaya, corn, blueberries, hard-boiled egg) *ad libitum*. Birds were maintained on the natural photoperiod and ambient temperatures (13–26°C) via a translucent roof and continuous air ventilation with the outdoors. Prior to experimentation, birds were given 10–15 days to acclimate to captivity and allow baseline CORT to return to close to naturally observed levels (Fischer et al., 2018).

Birds were randomly assigned to either a control or experimental CORT implant group (*N*=10 each per elevation). Sample sizes were chosen based on expected effect sizes and statistical power in similar laboratory studies on immunity (e.g. Hegemann et al., 2013; Schultz et al., 2017). On day 0, all birds received a 10 mm-long Silastic implant (1.47 mm inner diameter×1.96 mm outer diameter, Dow Corning Silastic silicone laboratory tubing, Fisher Scientific, Florence, KY, USA). CORT implants, for the experimental group, were filled with crystalline CORT (CORT implant for experimental group; Sigma-Aldrich C2505, Milwaukee, WI, USA), and sham implants, for the control group, were left empty, and all implants were sealed with A-100 Medical Silicone Adhesive (Factor II Inc., Lakeside, AZ, USA). This implant size and CORT dosage have been used to elevate baseline CORT moderately in adult passerines of similar mass (Newman et al., 2010; Shahbazi et al., 2014), and baseline CORT was measured before and after implant placement to confirm that baseline CORT was higher in birds with CORT than with sham implants (results below). Tubing was cleaned with 91% isopropyl alcohol and implants were assembled in a sterile hood. Assembled implants were soaked in sterile saline overnight and removed immediately before placement under the skin on the flank. The site of implantation was cleansed with 70% ethanol and lightly anesthetized with a topical lidocaine cream (Wound Gel & Pain Relief with 2% lidocaine, CVS, Woonsocket, RI, USA); a small incision in the skin was made using surgical scissors, the skin was gently separated from the underlying muscle, the implant was inserted in the opening and slid under the skin, and the incision was sealed with 3M Vetbond biological superglue (MWI Veterinary, Boise, ID, USA). Implants were removed by a reversal of this process 4 days after implantation; all implants still contained crystalline CORT upon removal. We chose an experimental period of 4 days because the implant technique generally elevates CORT for at least that length of time (e.g. Shahbazi et al., 2014). After a longer period, negative feedback can inactivate the HPA axis in birds, and this can lead to decreased endogenous CORT production, increased CORT clearance and lower overall circulating CORT levels (MacDougall-Shackleton et al., 2019; Vandenborne et al., 2005). To accommodate facility and personnel constraints, the experiment was run in eight rounds (controlled for in analyses, *N*=4–6 birds per round), each including equal numbers of control and experimental birds and involving both high- and low-elevation birds, from 18 March to 28 April 2019.

Blood samples were collected between 08:30 h and 10:00 h on days 0 (immediately before implant placement), 2 and 4 (before implant removal) of the experiment by pricking the alar vein with a sterile 26G needle and collecting blood into heparinized microhematocrit tubes. Baseline CORT samples were obtained within 3 min of entering the aviary to obtain baseline or near-baseline levels (Wingfield and Romero, 2010), and all other samples were collected within 10 min to minimize changes in immunity in response to capture (Zylberberg, 2015). Blood smears (details below) were produced immediately after sample collection. Remaining samples were stored on ice for no more than 2 h before being centrifuged for 5 min at 13,000 ***g*** (Unico PowerSpin MH Centrifuge, Dayton, NJ, USA). Plasma and packed cells were separated and stored at −30°C for a maximum of 9 months before use in hormone, immunity and malaria diagnostic assays.

### Hormone assays

Plasma CORT was quantified by radioimmunoassay as described and validated for this species (Names et al., 2021a). Reconstituted CORT was assayed in duplicate with tritiated CORT (Perkin Elmer NET399250UC, Waltham, MA, USA) and CORT antibody (MP Biomedicals 07120016, lot 3R3-PB, Solon, OH, USA) and combined with Ultima Gold scintillation fluid (Perkin Elmer 6013329) to be counted for 5 min or within 2% accuracy on a Beckman Coulter 6500 LS counter (Brea, CA, USA). Results were averaged across duplicates and corrected for individual sample recoveries. Mean (±s.d.) recovery was 92.85±2.94%, intra-assay variation (calculated using coefficient of variability, CV, between duplicates) and inter-assay variation (calculated using CV among assay standards) were 5.16% and 5.55%, respectively, the limit of detection was 9.36±0.26 pg (mean±s.d.) per tube, and the mean bound to free ratio was 0.35.

### Hemolysis–hemagglutination

We used the protocol described in Matson et al. (2005) to measure natural antibodies (agglutination score) and the complement enzyme cascade (hemolysis score) in plasma, with modifications by Zylberberg et al. (2013) to accommodate low sample volumes. Most samples were run in 10 µl volumes, but a subset of samples was run in 5 µl volumes (*N*=11/110, all reagents scaled accordingly) because of low sample volumes. All plates included a positive control (pooled chicken sample) and negative control (plasma replaced by 0.01 mol l^−1^ PBS). Samples were randomized across plates and scored blind to sample identity by one observer (G.R.N.). Our results are limited to agglutination score only, because complement activity was not high enough in Amakihi plasma to quantify hemolysis. Agglutination score was calculated as log_2_(*W*+1), where *W* was the last dilution well in which agglutination was detected.

### Circulating leukocytes and parasites

Circulating leukocytes and *Plasmodium* parasites were quantified by examining blood smears (Campbell, 1995). To produce smears, a drop of blood was spread into a thin film on a pre-cleaned glass slide, allowed to air dry, fixed using 100% methanol, and stained with Wright–Giemsa (Camco Stain Pak, Cambridge Diagnostic Products, Lauderdale, FL, USA). Smears were scored blind to sample identity by one observer (E.M.S.) for counts of leukocytes (lymphocytes, heterophils, monocytes, eosinophils, basophils) and *Plasmodium* parasites under 1000× magnification with oil immersion using the methods in Campbell (1995). To account for changes in hematocrit over the experimental period, the number of leukocytes and *Plasmodium* parasites per approximate erythrocyte count was multiplied by hematocrit volume to report leukocytes and parasites per milliliter of whole blood (continuous variables; Schultz et al., 2017). Because of some variation among slides, 100 good quality scoring ‘fields’ (representing approximately 100 erythrocytes each) did not exist for every slide. Only slides with a minimum of 20 good quality fields were scored (*N*=95) and of those slides, the mean (±s.e.m.) number of fields scored per slide was 62.2±2.69. To account for possible variation related to fields scored per slide, the parameter was included as a blocking variable in leukocyte and *Plasmodium* parasite models.

### Hematocrit

Packed red blood cell volume in centrifuged capillary tubes was measured blind to sample identity by one observer (G.R.N.) using a Critocaps micro-hematocrit capillary tube reader card.

### Malaria diagnostics

To determine avian malaria infection status (presence/absence), we used a modified version of the nested PCR method from Fallon et al. (2003) as described in Names et al. (2021a). Purified DNA was extracted from red blood cells using the Quick-DNA Miniprep Kit (Zymo Research, Irvine, CA, USA) following the manufacturer's protocols. Products from the second amplification were observed on 1.8% agarose gels. All reactions were run with a positive and negative control (malaria-infected Amakihi DNA and water, respectively). All samples were run twice and produced the same results in the two runs. The sensitivity of this assay is estimated at one parasite per 10^5^ red blood cells (Fallon et al., 2003).

### Statistical analyses

Data were analyzed with R Statistical Analysis Software version 3.5.0 (http://www.R-project.org/) using linear mixed models (LME, lme4 package). Baseline CORT and agglutination data were log transformed, leukocyte data were square root transformed, and *Plasmodium* data were cube root transformed to obtain normality of residuals; hematocrit data were normally distributed. The corrected Akaike's information criterion (AICc, MuMIn package) and Akaike weights (ω*_t_*, MuMIn package) were used to compare the series of possible models and the null model, and to choose the best-fit models among them (Burnham et al., 2011). All models within ΔAICc <2 of the lowest scoring model were considered statistically supported (Richards, 2005). Coefficient estimates, standard errors and 95% confidence intervals (CI, base R) for the best-fit models are reported. Parameter estimates with CIs that did not include zero were considered statistically significant. The list of tested models and coefficient estimates from the best-fit models are given in Tables S1 and S2, respectively. *Post hoc* analyses were conducted using Tukey's honestly significant difference (HSD) tests (emmeans package).

We tested for the effect of implant type (CORT, sham), experiment day (day 0, day 2, day 4) and bird elevation (high, low) or malaria infection status (infected, uninfected) and their interactions on five response variables: baseline CORT (ng ml^−1^), agglutination score, leukocytes per milliliter of blood, hematocrit and *Plasmodium* parasites per milliliter of blood. We included aviary room (A, B) and number of fields scored per slide (20–100, for leukocyte and *Plasmodium* models only) as blocking factors and individual bird identity nested within experimental round (1–8) as random effects. Hematocrit can decrease as a result of repeated blood sampling (Bradley et al., 2020), so to determine whether small differences in blood sampling volume affected our results, we tested whether total blood volume collected predicted hematocrit change during the experiment or varied by bird group (elevation or implant type). Total blood volume collected was not a good predictor of hematocrit change (estimate: 0.099±0.081, CI: −0.066 to 0.26), did not vary with elevation (high versus low – estimate: 2.51±3.05, CI: −3.69 to 8.70), and was only slightly higher (∼ 6 µl) in sham than in CORT implant birds (estimate: 6.39±3.05, CI: 0.20 to 12.58). We also tested for the effect of plasma volume per reaction on agglutination score and detected no significant effect (2.5 versus 5 µl – estimate: 0.018±0.039, CI: −0.058 to 0.094). As a result, total blood volume collected and plasma volume per reaction were excluded from subsequent analyses to avoid overparameterization. Monocytes, eosinophils and basophils were uncommon in slides (*N*=3/95, *N*=4/95 and *N*=0/95, respectively) and separate analyses of lymphocytes and heterophils per milliliter of blood produced the same supported models as analyses of pooled leukocytes, so we chose to present analyses of total leukocytes per milliliter of blood. Models including malaria infection as a predictor variable were run using only low-elevation bird data because of collinearity between malaria infection and elevation. All results are presented as means±s.e.m.

## RESULTS

### Baseline CORT

**Fig. 1.**
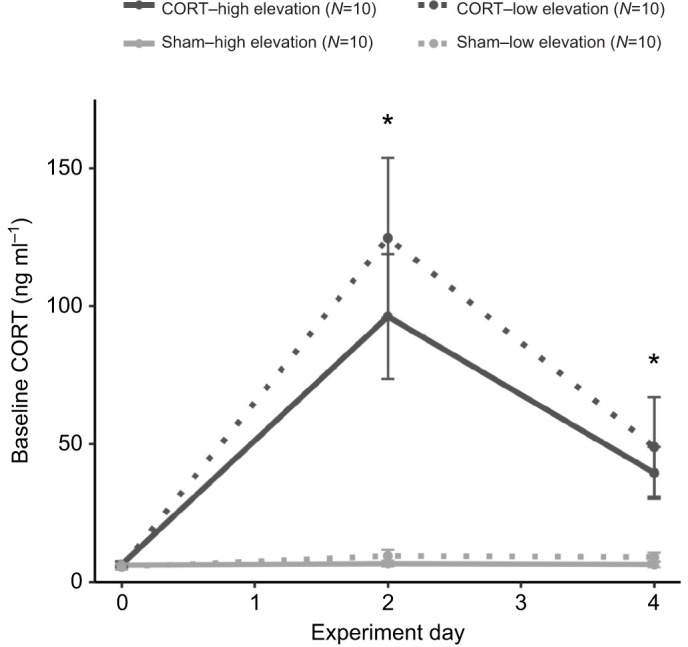
**Baseline corticosterone (CORT) across the experimental period in captive high- and low-elevation adult male Hawaii Amakihi (*Chlorodrepanis virens*) with CORT or sham implants.** Baseline CORT (collected within 3 min of disturbance) was higher in birds with CORT than with sham implants on day 2 (Tukey's HSD, estimate: 2.53±0.21, CI: 2.11 to 2.95) and day 4 (Tukey's HSD, estimate: 1.41±0.21, CI: 0.99 to 1.82). Asterisks indicate significant differences by implant on that experimental day. Values are presented as means±s.e.m. (sample sizes are indicated in parentheses).

Experiment day, implant type and their interaction were included in the best-fit model of baseline CORT in high- and low-elevation birds (*N*=120, ΔAICc=10.6). Baseline CORT was significantly higher in birds with CORT implants than with sham implants on day 2 (Tukey's HSD, estimate: 2.53±0.21, CI: 2.11 to 2.95) and day 4 (Tukey's HSD, estimate: 1.41±0.21, CI: 0.99 to 1.82) but not on day 0 (Tukey's HSD, estimate: 0.016±0.21, CI: −0.40 to 0.43, Fig. 1; see Fig. S2 for CORT levels of individual birds). In analyses including malaria infection status as a predictor variable of baseline CORT (low-elevation birds only), the same predictor variables were included in the best-fit model (*N*=60, ΔAICc=0.7), but the interaction among malaria infection status, experiment day and implant type was included in the next best supported model (*N*=60; Table S1). *Post hoc* analyses of the latter revealed that baseline CORT was significantly lower in infected than uninfected birds on day 0 (Tukey's HSD, estimate: −0.74±0.35, CI: −1.45 to −0.034).

### Immune indices

Neither implant type nor experiment day was a good predictor of agglutination score in high- and low-elevation birds or in only low-elevation birds (Table S1), and the best-fit model for both analyses was the null model (high  and low elevation: *N*=110, ΔAICc=2.6; low elevation: *N*=55, ΔAICc=2.3).

**Fig. 2.**
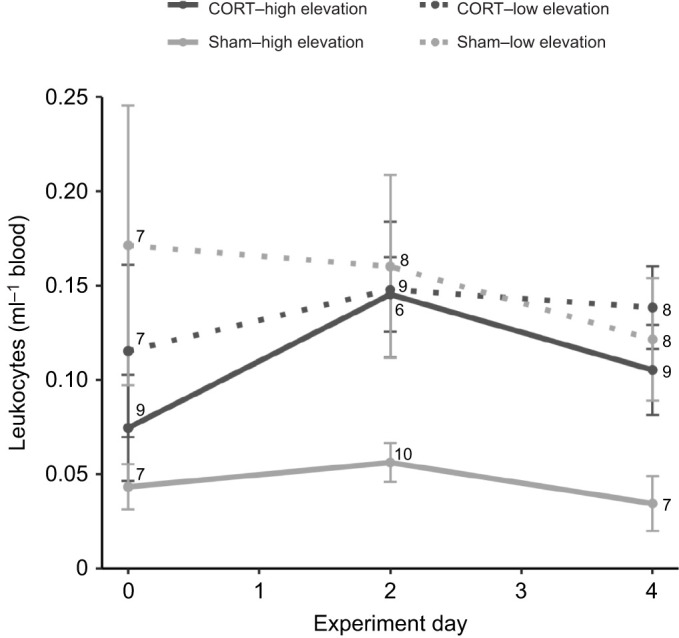
**Leukocyte levels across the experimental period in captive high- and low-elevation adult male Hawaii Amakihi with CORT or sham implants.** The number of leukocytes per milliliter of blood was greater in low- than in high-elevation birds (estimate: 0.080±0.035, CI: 0.014 to 0.15). Values are presented as means±s.e.m. (sample sizes are indicated on the figure).

Elevation was included in the best-fit model of leukocytes per milliliter of blood in high- and low-elevation birds (*N*=95, ΔAICc=0.5), and implant type and the interaction between implant type and elevation were also included in the next best supported model (*N*=95; Table S1). The number of leukocytes per milliliter of blood was significantly higher in low- compared with high-elevation birds (estimate: 0.080±0.035, CI: 0.014 to 0.15; Fig. 2). *Post hoc* analyses revealed an elevation-dependent effect of implant type, with leukocytes per milliliter of blood trending greater in high-elevation birds with CORT than with sham implants (Tukey's HSD, estimate: 0.095±0.048, CI: −0.0032 to 0.19) but not in low-elevation birds (Tukey's HSD, estimate: −0.011±0.048, CI: −0.11 to 0.086; Fig. 2). Malaria infection status, and not implant type, was included in the best-fit model of leukocytes per milliliter of blood in low-elevation birds (*N*=47, ΔAICc=1.9), and infected birds tended to have a higher leukocytes per milliliter of blood than uninfected birds (estimate: 0.067±0.045, CI: −0.026 to 0.16).

### Hematological health

**Fig. 3.**
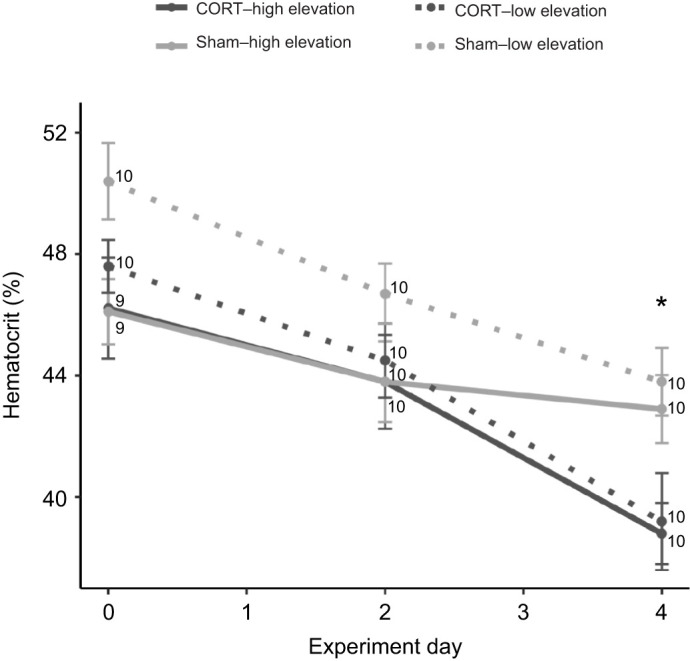
**Hematocrit across the experimental period in captive high- and low-elevation adult male Hawaii Amakihi with CORT or sham implants.** Hematocrit decreased over the experimental period for birds with CORT implants (Tukey's HSD, day 4 to 0 – estimate: −7.98±0.90, CI: −10.12 to −5.83) and sham implants (Tukey's HSD, day 4 to 0 – estimate: −5.15±0.90, CI: −7.29 to −3.00; Fig. 3). Hematocrit was lower in birds with CORT than with sham implants on day 4 (Tukey's HSD, estimate: −4.35±1.26, CI: −6.88 to −1.83). Asterisk indicates a significant difference by implant type on that experimental day. Values are presented as means±s.e.m. (sample sizes are indicated on the figure).

Hematocrit in high- and low-elevation birds was best predicted by a model that included implant type, experiment day and their interaction (*N*=118, ΔAICc=2.8). Hematocrit decreased over the experimental period for birds with both CORT implants (Tukey's HSD, day 4 to 0 – estimate: −7.98±0.90, CI: −10.12 to −5.83) and sham implants (Tukey's HSD, day 4 to 0 – estimate: −5.15±0.90, CI: −7.29 to −3.00; Fig. 3). However, hematocrit was significantly lower in birds with CORT than with sham implants by the end of the experimental period (Tukey's HSD, estimate: −4.35±1.26, CI: −6.88 to −1.83; Fig. 3). Implant type and experiment day were also included in analyses involving only low-elevation birds (*N*=60, ΔAICc=1.4). Again, hematocrit decreased over the experimental period (day 4 to 0 – estimate: −7.50±0.87, CI: −9.21 to −5.79) and was lower in birds with CORT than with sham implants (estimate: −3.02±1.17, CI: −5.39 to −0.80).

### Malaria parasitemia

**Fig. 4.**
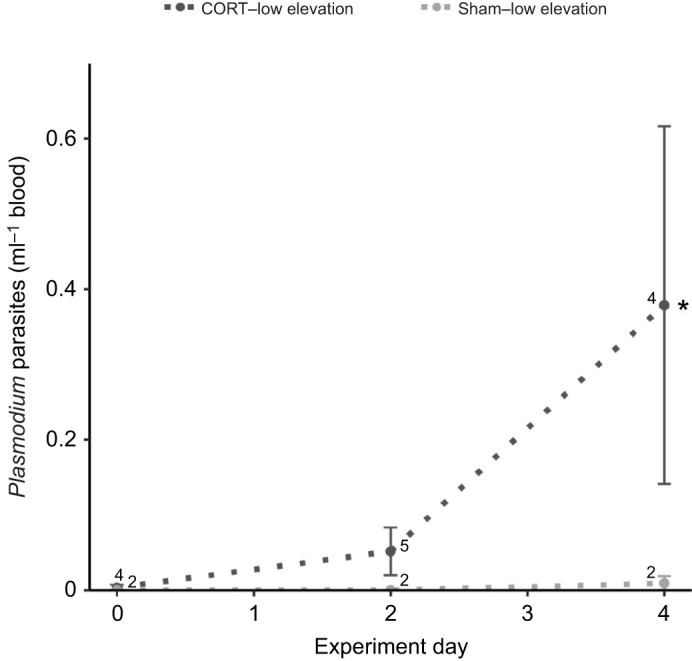
***Plasmodium* parasites across the experimental period in captive malaria-infected low-elevation adult male Hawaii Amakihi with CORT or sham implants.** The number of *Plasmodium* parasites per milliliter of blood was greater in birds with CORT than with sham implants (estimate: 0.50±0.14, CI: 0.25 to 0.76). Asterisk indicates a significant difference by implant type for all time points combined. Values are presented as means±s.e.m. (sample sizes are indicated on the figure).

A total of 8 low-elevation birds (*N*=6 CORT implant, *N*=2 sham implant) and 0 high-elevation birds tested positive for avian malaria via nested PCR at all three sampling points, and infection rates differed significantly by elevation (Pearson's chi-square: 47.53, d.f.: 1, CI: 0.29 to 0.51). *Plasmodium* parasites were detected via microscopy in a subset of blood smears from PCR-diagnosed infected birds (*N*=7/19) and in no blood smears from PCR-diagnosed uninfected birds (*N*=0/76). Implant type was included as a predictor of *Plasmodium* parasites per milliliter of blood in a statistically supported model that had nearly the same AICc as the best-fit model, the null model (ΔAICc=0.3). The number of *Plasmodium* parasites per milliliter of blood was significantly higher in birds with CORT than with sham implants (estimate: 0.50±0.14, CI: 0.25 to 0.76; Fig. 4).

## DISCUSSION

Our results partially support our hypothesis that chronically elevated CORT negatively affects Amakihi resilience to avian malaria, but our results do not provide support for suppression of innate immunity as the mechanism that links elevated CORT to lower resilience. As predicted, hematocrit was significantly lower in birds with CORT implants than with sham implants at the end of the experiment. Glucocorticoids can cause hematocrit to decrease through their effects on erythropoiesis and/or osmoregulation (Beck et al., 2016; Fair et al., 2007), although some studies have detected a positive relationship between CORT and hematocrit (Olanrewaju et al., 2007; Voorhees et al., 2013). Our results provide further support that elevated CORT is associated with declines in hematocrit in passerines. This relationship may have dire repercussions for Hawaiian honeycreepers threatened by avian malaria. During the acute phase of malaria infection, asexual *Plasmodium* parasites reproduce within erythrocytes and rupture host cells, leading to substantial reductions in hematocrit (up to 50% decrease) and anemia, which weakens birds and can increase mortality (LaPointe et al., 2012). As a result, Amakihi with both chronically elevated CORT and low hematocrit may be more susceptible to anemia and, subsequently, death as a result of malaria infection. Additionally, avian hematocrit can increase during specific life-history stages to support activities with increased metabolic demands, such as feeding of nestlings and investment in ornaments (Krause et al., 2016; Saino et al., 1997), though erythropoiesis may be constrained if resources are limited (Bradley et al., 2020). Consequently, chronically elevated CORT and its associated effects on hematocrit also have the potential to reduce Amakihi reproductive success through limits on aerobic performance during parental care or when conditions are challenging. Experimental investigations would help to test these predictions.

In further support of our hypothesis, circulating *Plasmodium* parasites were significantly more abundant in birds with CORT implants than with sham implants. Similar results were detected in house sparrows (*Passer domesticus*), where birds that received several days of CORT treatment via injections had greater *Plasmodium relictum* counts than control birds (Applegate, 1970). However, our results should be treated with caution as our sample sizes of malaria-infected birds were low. As malaria infection status was determined retrospectively via blood analysis after assigning birds to experimental groups, six infected birds were randomly assigned to the CORT group, while only two infected birds were assigned to the control group. Additional investigation will therefore be necessary to confirm this relationship. Regarding avian malaria diagnostic methods, our results confirmed that nested PCR is more effective at identifying avian malaria infections than microscopy. This is likely because the birds in our study were probably chronically, and not acutely, infected (we detected low *Plasmodium* parasite loads and most malaria-infected adult Hawaiian honeycreepers have low, chronic levels of infection; Samuel et al., 2015) and nested PCR is a more sensitive diagnostic technique than microscopy (Jarvi et al., 2002).

Although *Plasmodium* parasitemia was higher in birds with elevated CORT, the results from our immune assays did not support our hypothesis that suppression of innate immunity by CORT is the mechanism responsible for the relationship between CORT and parasite load. Natural antibodies did not vary with implant type or across the experimental period in either high- or low-elevation Amakihi. Relationships between CORT and natural antibodies are mixed in the literature. A negative correlation between natural antibodies and CORT was detected in fasted mallards (*Anas platyrhynchos*; Bourgeon et al., 2010), acute handling stress resulted in an increase in CORT and a decrease in natural antibodies in ring-billed gull chicks (*Larus delawarensis*; Chin et al., 2013) and in house sparrows (*Passer domesticus*; Gao et al., 2017), while no relationship between increased CORT and natural antibodies was detected in four of the five endemic Galápagos passerine species studied in Zylberberg (2015). Natural antibodies can control the early stages of viral and bacterial infections through multiple pathways (Ochsenbein et al., 1999), so it could be adaptive for natural antibodies in the Amakihi and Galápagos passerines to be maintained at a constant level, and kept relatively insensitive to modulation by hormones such as CORT. Alternatively, stress-induced immunosuppression of natural antibodies may occur in Amakihi but be unrelated to CORT-dependent mechanisms. In house sparrows in which endogenous CORT production was blocked, acute handling stress resulted in no change to CORT levels but still led to a decrease in natural antibodies, suggesting that CORT-independent mechanisms associated with the stress response are responsible for this immunosuppression (Gao and Deviche, 2019).

CORT also appeared to have no effect on circulating leukocytes in low-elevation birds, while leukocytes per milliliter of blood trended higher in high-elevation birds with CORT implants compared with those with sham implants. This relationship may be the result of a strong immunostimulatory effect of CORT in the first hours of the experiment. Shortly after an increase in CORT, heterophils are mobilized, accumulate in the blood, and enter a higher state of activation, which can result in a temporary overall increase in circulating leukocytes (Koutsos and Klasing, 2014; McRee et al., 2018), whereas chronically high CORT can result in reduced leukocyte activity and an eventual decrease in circulating leukocytes (Koutsos and Klasing, 2014; Shini et al., 2010). In high-elevation Amakihi, circulating leukocytes increased during the first 48 h of the CORT treatment and then decreased between day 2 and 4 (Fig. 2A), suggesting that an acute increase in CORT had an immunostimulatory effect on leukocytes in high-elevation Amakihi, while several days of CORT treatment may have an immunosuppressive effect. More frequent sampling, particularly during the first hours of CORT treatment, would help to parse out these divergent effects. Finally, while we did not detect a relationship between elevated CORT and immunosuppression, we were only able to measure two aspects of innate immunity because the amount of blood that could be collected from these small birds (average mass: 12 g) was limited. As a result, suppression of innate immunity should not be ruled out as a mechanism linking elevated CORT to increased malaria susceptibility until the effects of elevated CORT on other components of innate immunity (e.g. acute phase proteins, lysozymes) have been explored.

Overall, low-elevation birds maintained higher leukocytes per milliliter of blood compared with high-elevation birds. This result may suggest that low-elevation Amakihi, which are known to be more resilient to avian malaria (Atkinson et al., 2013), are less responsive to the stimulatory and suppressive effects of elevated CORT. In environments where the risk of infection is consistently high (i.e. low-elevation habitats for native birds in Hawaii), it would be adaptive for hosts to maintain a heightened level of immune activity if the energetic costs of immunity are outweighed by increased survival in the event of infection (Koutsos and Klasing, 2014; Martin et al., 2008). The activation of inflammatory cytokines during the first hours of malaria infection can control and help to predict the course of infection, and the circulating leukocytes most prevalent in our Amakihi samples (lymphocytes and heterophils) both produce pro-inflammatory cytokines and chemokines (Artavanis-Tsakonas et al., 2003; Cornet and Sorci, 2014; Lee et al., 2006). This evidence, together with our finding that the number of leukocytes per milliliter of blood was greater overall in low- than in high-elevation Amakihi, could suggest a role of leukocytes in mediating the response to avian malaria infections, although experimental infections would be necessary to test this possibility.

Prior to experimental treatment, baseline CORT was initially lower in infected than in uninfected birds. This relationship was surprising because baseline CORT in free-living Amakihi does not differ in infected versus uninfected birds, even in the same populations studied here (Names et al., 2021a). Environmental variables, such as weather and food availability, can have significant effects on CORT levels (Romero and Wingfield, 2016) and presumably vary for free-living birds. Controlling for these and other variables for captive birds appears to have revealed a relationship between baseline CORT and malaria infection status that was masked among birds in the field. Further investigations would help to determine whether baseline CORT is lower as a result of infection, or whether downward modulation of baseline CORT in infected birds contributes to avian malaria resilience.

Finally, we found that Silastic implants are an effective and safe way to modify hormone levels in Amakihi. Experimental hormone manipulation has not previously been attempted in any species of Hawaiian honeycreeper. CORT implants significantly increased circulating CORT above baseline levels and above the levels of birds with sham implants. Further, the implant size tested here delivered physiologically relevant doses of CORT. However, we did detect some individual variability in response to implants (variance: 0.14, s.d.: 0.38; Fig. S2). Many individuals experienced an increase in baseline CORT that was within the biological range observed in free-living Amakihi males on Hawaii Island (range upper bound: 116.07 ng ml^−1^; Names et al., 2021a). Baseline CORT in a few individuals increased to pharmacological levels, particularly on experiment day 2, but these levels were still within the upper range observed in other songbird species (e.g. Addis et al., 2011).

Chronically elevated glucocorticoids can be immunosuppressive and may reduce a host's ability to resist or cope with infectious diseases (Fonner et al., 2017; Gervasi et al., 2017; Sapolsky et al., 2000). The goal of this study was to explore how elevated CORT (the main avian glucocorticoid) influences Amakihi resilience to avian malaria through its effects on innate immunity, hematocrit and *Plasmodium* parasite load. CORT implants resulted in a decrease in hematocrit and an increase in *Plasmodium* parasitemia, suggesting that chronically elevated CORT may cause Amakihi to be less resilient to avian malaria infection. Suppression of innate immunity (natural antibodies, leukocytes) by elevated CORT does not seem to be the mechanism by which CORT affects avian malaria resilience, at least on the time scale tested here (4 days of treatment), but additional research involving other aspects of innate immunity is needed. These findings highlight the importance of considering physiology in the context of emerging infectious diseases and raise new questions regarding relationships between glucocorticoids and disease susceptibility.

## Supplementary Material

Supplementary information
