# Supplementary material for: Stress in paradise: effects of elevated corticosterone on immunity and avian malaria resilience in a Hawaiian passerine
Source: J Exp Biol. 2021 Oct 19;224(20):jeb242951. doi: 10.1242/jeb.242951 (PMC8546672; doi:10.1242/jeb.242951)
Supplement: Supplementary information [file jexbio-224-242951-s1.pdf]

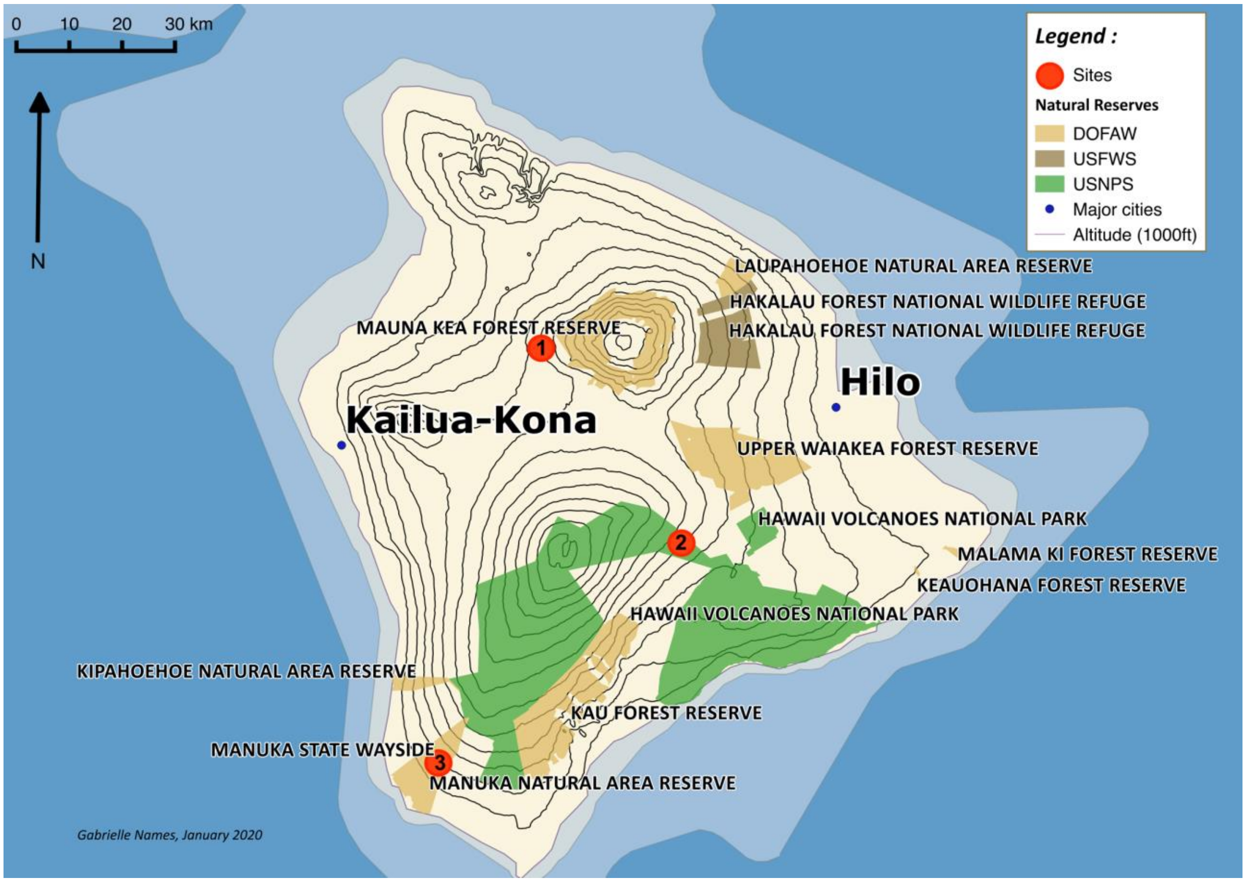

Fig. S1. Map of field sites (red points) on Hawaii Island, Hawaii, USA (1 – Kaohe Game Management Area, 2 – Hawaii Volcanoes National Park, 3 – Manuka State Wayside Park). Major cities, Hawaii Department of Forestry and Wildlife Forest Reserves (DOFAW), United States National Parks (USNPS), and United States Fish and Wildlife Service Reserves (USFWS) are included as points of reference.

Journal of Experimental Biology • Supplementary information

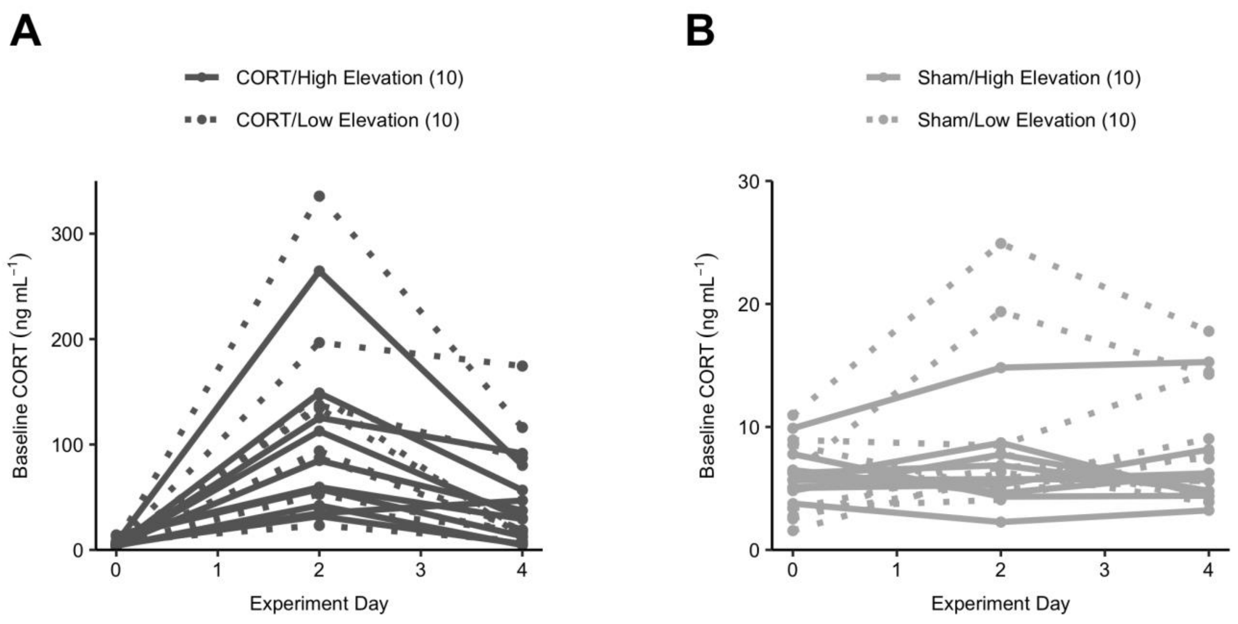

Fig. S2. Baseline corticosterone (CORT, collected within 3 min of disturbance) across the experimental period in individual captive high- and low-elevation adult male Hawaii Amakihi (*Chlorodrepanis virens*) (A) with CORT implants, and (B) with sham implants.

Table S1. List of models (ranked in order of lowest to highest AICc score) predicting baseline corticosterone (CORT), agglutination score, leukocytes (per milliliter of blood), hematocrit, and *Plasmodium* parasites (per milliliter of blood) in captive high- and low-elevation or only low elevation adult male Hawaii Amakihi (*Chlorodrepanis virens*).

Click here to download Table S1

Table S2. Model estimates and 95% CIs for the best-supported models predicting baseline corticosterone (CORT), agglutination score, leukocytes (per milliliter of blood), hematocrit, and *Plasmodium* parasites (per milliliter of blood) in captive high- and low-elevation or only low-elevation adult male Hawaii Amakihi (*Chlorodrepanis virens*).

Click here to download Table S2

Journal of Experimental Biology • Supplementary information
